# Transcriptome-Wide m^6^A Methylation in Skin Lesions From Patients With Psoriasis Vulgaris

**DOI:** 10.3389/fcell.2020.591629

**Published:** 2020-11-05

**Authors:** Ya-Nan Wang, Hong-Zhong Jin

**Affiliations:** Department of Dermatology, Peking Union Medical College Hospital, Chinese Academy of Medical Sciences & Peking Union Medical College, Beijing, China

**Keywords:** N6-methyladenosine, m^6^A methylation, psoriasis vulgaris, MeRIP-seq, RNA modification

## Abstract

N^6^-methyladenosine (m^6^A) methylation, as the most prevalent internal RNA modification, has been revealed to play critical roles in various biological functions. In this study, we performed m^6^A transcriptome-wide profiling in three kinds of skin tissue: involved psoriatic skin (PP), uninvolved psoriatic skin (PN), and healthy control skin samples (NN). The findings revealed that transcripts of PP contained the fewest m^6^A peaks and lowest m^6^A peak density. The greatest differences of m^6^A methylation were observed in the PP vs. NN and PP vs. PN comparisons. Intriguingly, in these comparisons, hypermethylated m^6^A was mainly enriched within the CDSs and 3′UTRs, while hypomethylated m^6^A was not only enriched within CDSs and 3′UTRs, but also within 5′UTRs. GO and KEGG pathway analyses indicated that hypermethylated transcripts in PP were particularly associated with response-associated terms, cytokine production, and olfactory transduction. Meanwhile, hypomethylated transcripts in PP were mainly associated with development-related processes and the Wnt signaling pathway. In addition, we discovered that 19.3–48.4% of the differentially expressed transcripts in psoriasis vulgaris were modified by m^6^A, and that transcripts with lower expression were more preferentially modified by m^6^A. Moreover, upregulation of gene expression was often accompanied by upregulation of m^6^A methylation, suggesting a regulatory role of m^6^A in psoriasis vulgaris gene expression.

## Introduction

Psoriasis is a chronic, systemic, inflammatory disease, affecting about 2% of the world’s population (Greb et al., 2016). The most common subtype is psoriasis vulgaris, accounting for 80–90% of all cases (Greb et al., 2016). In 2014, the WHO adopted a resolution that defines psoriasis as a chronic, non-communicable, painful, disfiguring, and disabling disease for which there is no cure (World Health Organization, 2014). The skin lesions of psoriasis vulgaris are characterized by excessive proliferation and abnormal differentiation of keratinocytes, accompanied by significant infiltration of inflammatory cells, which originate as a result of dysregulation of immunity triggered by environmental and genetic stimuli (Boehncke and Schon, 2015; Hugh and Weinberg, 2018). Situated between heredity and the environment are epigenetic markers, which are another layer of biological information (Pollock et al., 2017). Extensive epidemiological and molecular analyses have provided evidence for the involvement of epigenetics in psoriasis vulgaris (Pollock et al., 2017; Rendon and Schäkel, 2019). The effects of pharmacological inhibitors of epigenetic-modifying enzymes in psoriasis vulgaris have been studied and verified (Bovenschen et al., 2011; Hammitzsch et al., 2015). Epigenetic mechanisms modify gene expression without changing the sequence of the genome, such as long non-coding RNA (lncRNA) and microRNA (miRNA) silencing, and DNA methylation, but RNA modifications in psoriasis vulgaris have not yet been reported (Rendon and Schäkel, 2019).

In the past, the study of epigenetic changes in human diseases mainly focused on DNA methylation and histone modification. Although post-transcriptional modifications of RNA have been known for more than 50 years, the effects of these modifications on the regulation of gene expression has only begun to be explored in recent years due to the previous lack of sensitive detection techniques (He, 2010). Against the background of the rapid development of second-generation sequencing technology, as early as 2012, the sequencing technology of methylated RNA immunoprecipitation with next-generation sequencing (MeRIP-Seq) was developed (Dominissini et al., 2013). m^6^A (N6-methyladenosine), involving methylation at the 6th position nitrogen atom of adenine (A) of RNA, is the most prevalent RNA modification in messenger RNA (mRNA) and lncRNA of higher eukaryotes (Wang et al., 2017b; Wang et al., 2020). m^6^A methylation is highly conserved and occurs widely in eukaryotic species ranging from yeast, plant, and drosophila to mammals, as well as viruses (Bi et al., 2019). m^6^A methylation can be “written” by methyltransferases [e.g., methyltransferase-like 3 (METTL3), methyltransferase-like 14 (METTL14), and Wilms tumor 1-associated protein (WTAP)] and “erased” by demethylases [e.g., fat-mass and obesity-associated protein (FTO) and alkB homolog 5 (ALKBH5)], which confirms that m^6^A methylation is dynamic and reversible (Bi et al., 2019). In addition, the discovery of binding proteins [e.g., YTH domain family 1–3 (YTHDF1–3)] confirmed that m^6^A methylation has a wide range of biological effects and significance (Fu et al., 2014). In recent years, m^6^A methylation has emerged as a critical post-transcriptional regulator of gene expression programs, and it can regulate various aspects of RNA, including splicing, transport, translation, and stability (Fu et al., 2014; Frye et al., 2018). It has been found that m^6^A methylation is closely related to metabolism, carcinogenesis, nervous system, mental, oral, and immune diseases (Church et al., 2010; Engel and Chen, 2018; Pan et al., 2018). However, to the best of our knowledge, no reports have been published on m^6^A methylation in the context of psoriasis vulgaris.

In this study, we established a transcriptome-wide m^6^A methylome profile of psoriasis vulgaris, as assessed by MeRIP-Seq. In addition, we used RNA-Seq data to perform a combined analysis of m^6^A methylation and mRNA levels.

## Materials and Methods

### Patient Samples

Four patients with psoriasis vulgaris were recruited from the outpatient clinics of the Peking Union Medical College Hospital (Supplementary Table 1 and Supplementary Figure 1). Prior to recruitment, none of the patients had received systemic tretinoin, glucocorticoid, immunosuppressant, or biological agents, nor had they received PUVA/solarium/UV treatment for at least 3 months or topical therapy for at least 2 weeks before the study start. Infectious diseases, tumors, autoimmune diseases, and other immune-related diseases were ruled out. The subjects had no coagulatory disorders or other diseases making them unsuitable to undergo a surgical operation. Skin biopsies were obtained from the patients with psoriasis vulgaris by minimally invasive surgery under aseptic conditions with local anesthesia (0.5% lidocaine). Two types of skin sample were collected from each patient: one from active lesions and the other from skin not exhibiting any of the macroscopic changes related to psoriatic lesions, at least 3 cm away from the active lesions. Four age- and sex-matched healthy controls without a personal or family history of psoriasis vulgaris were enrolled from the Department of Plastic Surgery at Peking Union Medical College Hospital. Healthy skin tissues were obtained from these volunteers. This study was approved by the Ethics Committee of Peking Union Medical College Hospital and informed consent was obtained from all patients and unaffected individuals.

### MeRIP-Seq, RNA-Seq, and Data Analysis

The MeRIP-Seq was performed by Cloudseq Biotech Inc. (Shanghai, China), in accordance with the published procedure with slight modifications (Meyer et al., 2012). Briefly, four biological replicates were used for the PP, PN, and NN groups. Total RNA was extracted from the three groups of skin tissue using TRIzol reagent (Life Technologies, Carlsbad, CA, United States). The quality and quantity of total RNA were assessed using NanoDrop ND-2000 (Thermo Fisher Scientific, Waltham, MA, United States). The RNA integrity was measured using denaturing agarose gel electrophoresis. Seq-Star^TM^ poly(A) mRNA Isolation Kit (Arraystar, Rockville, MD, United States) was used to isolate mRNA from total RNA. mRNA was randomly fragmented to 200 nt by RNA Fragmentation Reagents (Ambion) (Invitrogen, Carlsbad, CA, United States). A total of 5 μg of fragmented mRNA was saved as input control for RNA-Seq, while 500 μg of fragmented mRNA was used to perform m^6^A RNA immunoprecipitation with GenSeq^TM^ m^6^A-MeRIP Kit (GenSeq, Beijing, China) [including anti-m^6^A polyclonal antibody (Synaptic Systems, Goettingen, Germany)]. Both the m^6^A IP sample and the input sample (without immunoprecipitation) were used for library preparation with NEBNext^®^ Ultra II Directional RNA Library Prep Kit (New England Biolabs, Ipswich, MA, United States). The library quality was evaluated with BioAnalyzer 2100 system (Agilent Technologies, Santa Clara, CA, United States). Library sequencing was performed on an Illumina HiSeq instrument with 150 bp paired-end reads.

Paired-end reads were harvested from the Illumina HiSeq 4000 sequencer and were subjected to quality control by Q30. After 3′ adaptor-trimming and the removal of low-quality reads by cutadapt software (v1.9.3) (Martin, 2011), the reads were aligned to the reference genome (UCSC HG19) with Hisat2 software (v2.0.4) (Kim et al., 2015). Methylated sites on RNAs (peaks) were identified using Model-based Analysis of ChIP-Seq (MACS) software (Zhang et al., 2008). Peak numbers refer to peaks present in all four samples in each group. Differentially methylated sites (fold change > 2 and *P* < 0.00001) on mRNAs were identified by diffReps (Shen et al., 2013). Genes of interest were visualized in the IGV (Integrative Genomics Viewer) software (v2.3.68) (Thorvaldsdóttir et al., 2013). Those peaks identified by both software programs overlapping with exons of mRNA were determined and selected using custom-made scripts. The GO analysis and pathway enrichment analysis were performed on the differentially methylated protein-coding genes by using GO^1^ and KEGG databases^2^. Sequence motifs were identified using Homer (Heinz et al., 2010). Gene expression was calculated by Cufflinks (Trapnell et al., 2012) using sequencing reads from input samples. Cuffdiff (Trapnell et al., 2012) was used to find DE genes.

### MeRIP-RT-qPCR

RNA fragmentation and m^6^A-immunoprecipitation were performed as described above. Briefly, total RNA was extracted using TRIzol (Life Technologies) and fragmented by RNA fragmentation reagents (Thermo Fisher Scientific) or not. After saving 50 ng of the total RNA as input, the remaining RNA (2 μg) was used for m^6^A-immunoprecipitation with anti-m^6^A polyclonal antibody (Synaptic Systems) in 500 μL of IP buffer (150 mM NaCl, 0.1% NP-40, 10 mM Tris, pH 7.4, 100 U RNase inhibitor) to obtain the m^6^A pull-down portion (m^6^A IP portion). m^6^A RNAs were immunoprecipitated with Dynabeads^®^ Protein A (Thermo Fisher Scientific) and eluted twice with elution buffer (5 mM Tris-HCL pH 7.5, 1 mM EDTA pH 8.0, 0.05% SDS, 20 mg/ml Proteinase K). m^6^A IP RNAs were recovered by ethanol precipitation and RNA concentration was measured with Qubit^®^ RNA HS Assay Kit (Thermo Fisher Scientific). Then, 2 ng of the total RNA and m^6^A IP RNA were used to synthesize complementary DNA by using the iScript cDNA Synthesis Kit (Bio-Rad, CA, United States). Real-time PCR was subsequently performed, using an SYBR Premix Ex Taq (Takara, Liaoning, China) and ABI 7500 Sequence Detection System (Thermo Fisher Scientific). All procedures were performed in accordance with the manufacturer’s protocols. The sequences of the primers used are presented in Supplementary Table 2.

### Statistical Analyses

Experiments were performed at least three times and representative results are shown. Statistical analysis was performed using GraphPad Prism Version 8.0 software. Differences between individual groups were analyzed using the chi-squared test and Student’s *t*-test (two-tailed and unpaired) with triplicate or quadruplicate sets. Pearson’s correlation was adopted to carry out the correlation analysis. A value of *P* < 0.05 was considered statistically significant.

## Results

### Overview of m^6^A Methylation in Psoriasis Vulgaris mRNA

To obtain the transcriptome-wide m^6^A map of psoriasis vulgaris, we examined three kinds of skin tissue, namely, involved psoriatic skin (PP), uninvolved psoriatic skin (PN), and healthy control skin samples (NN), using m^6^A-targeted antibody coupled with high-throughput sequencing (i.e., MeRIP-Seq). Psoriasis Area and Severity Index (PASI) scores for psoriatic patients ranged from 10.8 to 16.7. Using Illumina HiSeq 4000, we acquired 68,678,097, 62,087,334, and 62,121,508 reads from PP, PN, and NN, respectively. After end-trimming and quality filtering, 57,334,158, 45,130,912, and 49,749,336 high-quality reads (83.11, 72.65, and 80.04% of the total reads) from PP, PN, and NN, respectively, were mapped to the human reference genome (UCSC HG19) (Supplementary Table 3).

**FIGURE 1:**
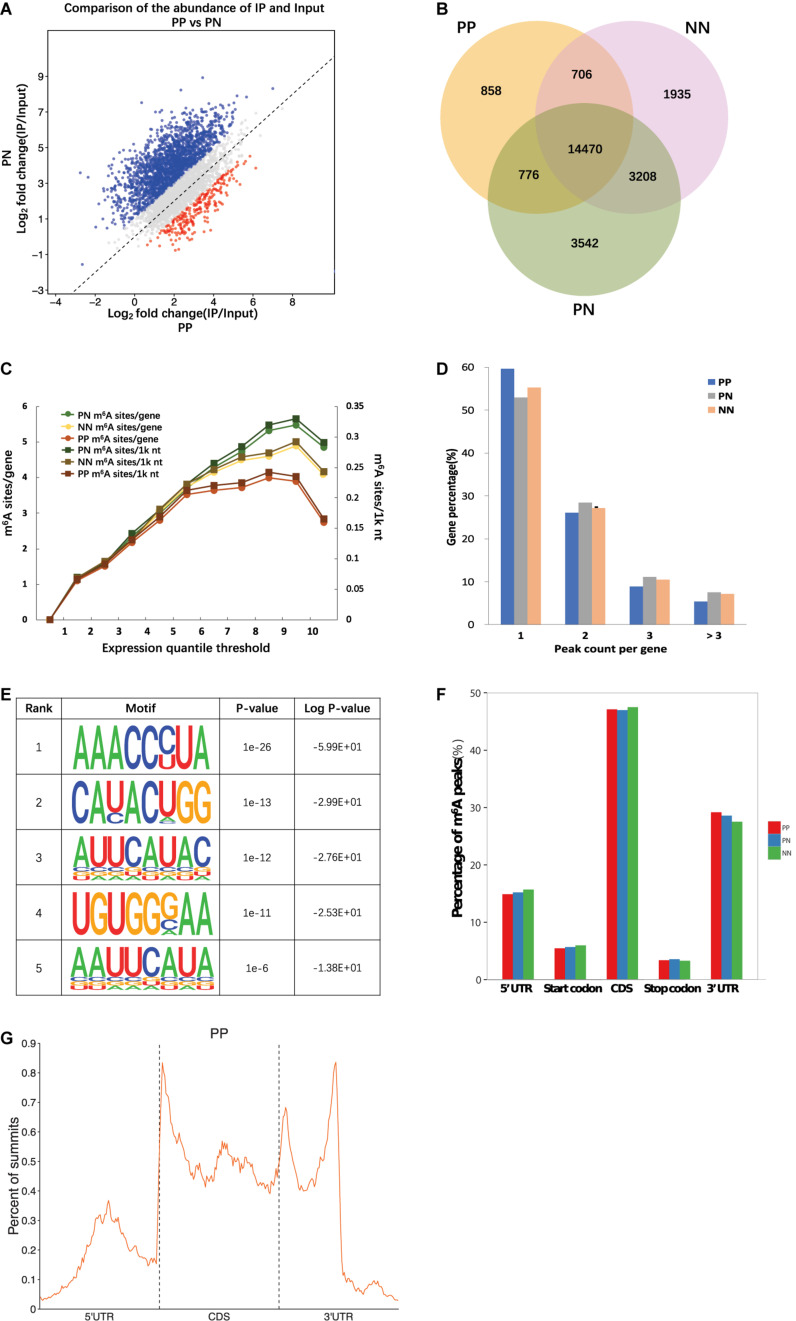
Overview of m^6^A methylome in psoriatic vulgaris skin. **(A)** Comparison of the number of m^6^A peaks identified in PP and PN. **(B)** Numbers of group-specific and common m^6^A peaks. **(C)** Estimation of m^6^A density on transcripts. Transcripts with different expression level are divided to 10 groups, and the m^6^A density of PP, PN, and NN samples is calculated separately. (**D**) Percentage of m^6^A methylated genes with different m^6^A peak number. **(E)** The top five motifs enriched across m^6^A peaks identified from involved psoriatic skin samples. **(F)** Distribution of m^6^A peaks across the length of mRNAs. 5′UTRs, CDS, and 3′UTRs of PP mRNAs are individually binned into regions spanning 1% of their total length, and the percentage of m^6^A peaks that fall within each bin is determined. **(G)** The distribution of m^6^A peak from PP along a metagene.

The fewest m^6^A peaks (sites) were identified in PP, while the most were identified in PN. Specifically, 16,868 m^6^A peaks were identified from 16,520 genes in PP, 22,144 m^6^A peaks were identified from 17,665 genes in PN, and 20,408 m^6^A peaks were identified from 17,358 genes in NN (Figure 1A, Supplementary Figure 2, Supplementary Table 4, and Supplementary Data 1). We next compared the shared peaks between groups; we found that transcripts of PP and NN carried the fewest shared m^6^A peaks (a total of 15,176 m^6^A peaks from 10,604 genes), while transcripts of PN and NN carried the largest number of shared m^6^A peaks (a total of 17,678 m^6^A peaks from 11,816 genes), suggesting that the difference between PP and NN was greater than that between PN and NN (*P* < 0.001) (Figure 1B).

### m^6^A Peak Density and Distribution Pattern in Psoriasis Vulgaris mRNA

Based on the above results, we estimated that there were 0.62–0.75 m^6^A peaks per 1,000 nucleotides or 1.02–1.25 m^6^A peaks per identified transcript in PP, PN, and NN. Among them, transcripts of PP contained the lowest m^6^A peak density (0.62/1k nt or 1.02/gene), while transcripts of PN contained the highest (0.75/1k nt or 1.25/gene) (Figure 1C and Supplementary Table 4). The numbers of m^6^A peaks varied widely among individual genes, and most of them contained a single m^6^A peak. Specifically, 59.68, 52.95, and 55.25% of the methylated transcripts contained a single m^6^A peak in PP, PN, and NN, while 26.07, 28.42, and 27.15% of the methylated transcripts contained two m^6^A peaks per mRNA, respectively. For three m^6^A peaks, the percentages were further reduced to 8.88, 11.13, and 10.46% in PP, PN, and NN, respectively. Only 5.38, 7.50, and 7.14% of the methylated transcripts containing more than three m^6^A peaks (Figure 1D). To determine whether the identified m^6^A peaks were enriched at consensus sequences of RRACH (R represents purine, A is m^6^A, and H is a non-guanine base), we performed motif analysis and found consistent results in PP, PN, and NN (Figure 1E and Supplementary Figure 3). To analyze the distribution pattern of m^6^A in psoriasis vulgaris, the metagene profiles of all identified m^6^A peaks were investigated in the transcriptomes of PP, PN, and NN, which revealed that m^6^A peaks were highly enriched within the coding sequences (CDSs) and 3′ untranslated regions (3′UTRs) (Figures 1F,G and Supplementary Figure 4). These features of m^6^A methylation indicate its high conservation in psoriasis vulgaris.

### Analysis of Differentially Methylated RNAs Among PP, PN, and NN Samples

**FIGURE 2:**
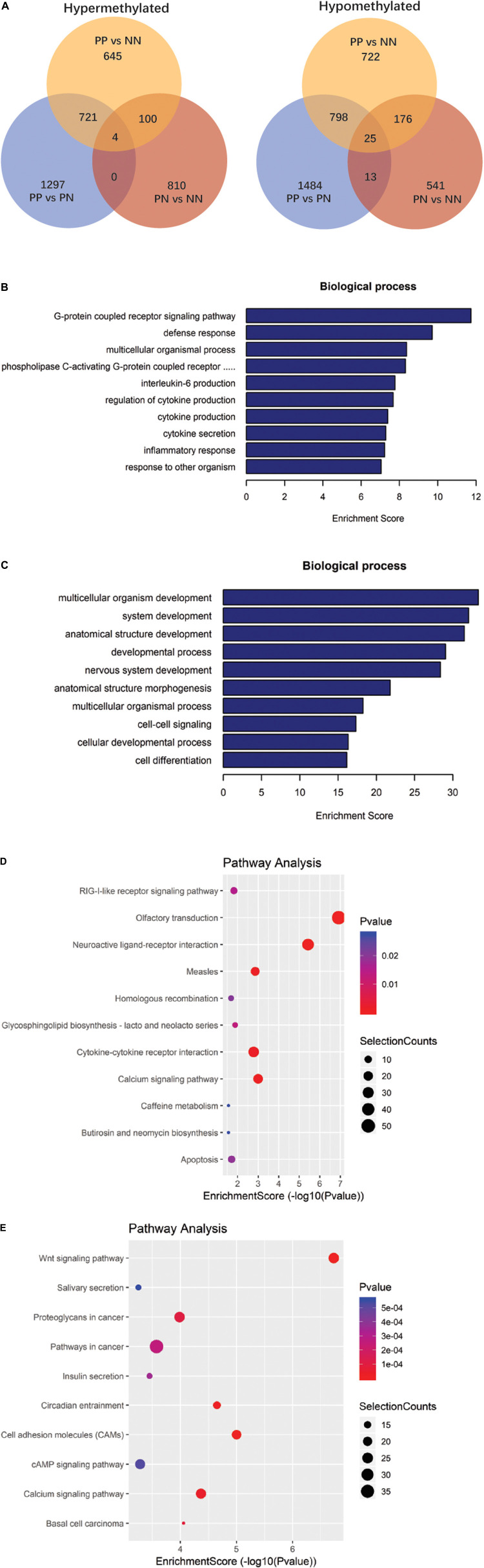
Gene ontology and Kyoto Encyclopedia of Genes and Genomes analyses of coding genes containing altered m^6^A peaks. **(A)** Comparison of the number of hypermethylated and hypomethylated m^6^A peaks identified in PP, PN, and NN samples. **(B)** The top 10 gene ontology terms were significantly enriched for the hypermethylated genes in PP vs. NN. **(C)** The top 10 gene ontology terms were significantly enriched for the hypomethylated genes in PP vs. NN. **(D)** The top 10 significantly enriched pathways for the hypermethylated genes in PP vs. NN. **(E)** The top 10 significantly enriched pathways for the hypomethylated genes in PP vs. NN.

We next analyzed the differentially methylated RNAs (DMRs). The greatest differences were observed in the PP vs. NN and PP vs. PN comparisons (Figure 2A, Supplementary Table 5, and Supplementary Data 2). In the PP vs. NN and PP vs. PN comparisons, the number of hypomethylated m^6^A peaks was much greater than that of hypermethylated m^6^A peaks in PP (*P* < 0.001). Specifically, compared with NN, transcripts of PP contained more hypomethylated m^6^A peaks (1,719 m^6^A peaks from 1,113 genes) than hypermethylated ones (1,470 m^6^A peaks from 1,127 genes). When compared with PN, transcripts of PP also contained more hypomethylated m^6^A peaks (2,316 m^6^A peaks from 1,568 genes) than hypermethylated ones (2,024 m^6^A peaks from 1,362 genes) (Figure 2A and Supplementary Table 5). However, when compared with NN, PN contained slightly more hypermethylated m^6^A peaks (914 m^6^A peaks from 691 genes) than hypomethylated ones (755 m^6^A peaks from 537 genes), but this difference was relatively small compared with those of PP vs. NN and PP vs. PN (*P* < 0.001) (Figure 2A and Supplementary Table 5). Furthermore, upon analyzing the m^6^A peak distribution pattern of these DMRs in PP vs. NN, PP vs. PN, and PN vs. NN, we found that the hypermethylated m^6^A peaks were mainly enriched within CDSs and 3′UTRs, while hypomethylated m^6^A peaks were not only enriched within CDSs and 3′UTRs, but also highly enriched within 5′UTRs (Table 1).

**TABLE 1:** Number of m^6^A peaks among PP, PN, and NN samples.

	PP vs. NN (up-regulated)	PP vs. NN (down-regulated)	PP vs. PN (up-regulated)	PP vs. PN (down-regulated)	PN vs. NN (up-regulated)	PN vs. NN (down-regulated)
5′UTR	92	208	139	407	77	76
Start codon	36	73	54	126	34	37
CDS	628	802	882	973	367	329
Stop codon	70	56	112	81	36	29
3′UTR	382	286	572	304	220	190

### Gene Ontology Analysis of Differentially Methylated RNAs Among PP, PN, and NN Samples

To deduce the potential biological significance of m^6^A methylation in psoriasis vulgaris, we analyzed these DMRs by performing Gene Ontology (GO) analysis. GO analysis revealed that, compared with PN or NN, hypermethylated DMRs in PP were particularly associated with response-related items [e.g., defense response, response to other organisms, inflammatory response, and response to (external) biotic stimulus] and cytokine-related items (e.g., regulation of cytokine production, cytokine production, and interleukin-6 production), suggesting that these hypermethylated DMRs may be involved in the response to environmental stimulations at the initial stage of psoriasis vulgaris (Figure 2B, Supplementary Figure 5A, and Supplementary Data 3). Meanwhile, hypomethylated DMRs in PP were mainly enriched in development-related items (e.g., multicellular organism development, system development, anatomical structure development, developmental process, and nervous system development) and cell–cell signaling, which suggested that the function of hypomethylated DMRs differed from that of hypermethylated DMRs. Hypomethylated DMRs in PP were mainly focused on cell and tissue development processes (Figure 2C, Supplementary Figure 5B, and Supplementary Data 3). In addition to these shared gene ontological terms, DMRs in the PP vs. NN and PP vs. PN comparisons were also associated with some specific terms. For example, in PP vs. NN, hypermethylated DMRs in PP were specifically associated with the G-protein-coupled receptor signaling pathway (Figure 2B and Supplementary Data 3), including cannabinoid receptors that are involved in the proliferation/differentiation and immune activity of keratinocytes, indicating that these hypermethylated DMRs may participate in critical processes of psoriasis vulgaris (Tóth et al., 2019). Meanwhile, hypomethylated DMRs in PP were specifically associated with the polyol metabolic process (Figure 2C and Supplementary Data 3), which may be related to the previous epidemiological finding that psoriasis patients tended to suffer from metabolic diseases (Hu et al., 2019). When compared with PN, hypermethylated DMRs in PP were specifically associated with cell cycle-related items (e.g., mitotic cell cycle process, mitotic cell cycle, cell cycle, and mitotic nuclear division) (Supplementary Figure 5A and Supplementary Data 3), while hypomethylated DMRs in PP were specifically associated with negative regulation of cellular component movement, locomotion, and learning (Supplementary Figure 5B and Supplementary Data 3). Given that the cell cycle-related genes were dysregulated in keratinocytes in psoriasis vulgaris, m^6^A methylation should play an important role in dysregulation of the cell cycle in keratinocytes in psoriasis vulgaris (Pasquali et al., 2019). Besides, PN and NN exhibited various differences, and the DMRs were mainly associated with system process and multicellular organismal process (Supplementary Figures 5C,D and Supplementary Data 3). All of these results suggest that m^6^A methylation participates in various pathophysiological aspects of psoriasis vulgaris.

### Kyoto Encyclopedia of Genes and Genomes Pathway Analysis of Differentially Methylated Genes Among PP, PN, and NN Samples

**FIGURE 3:**
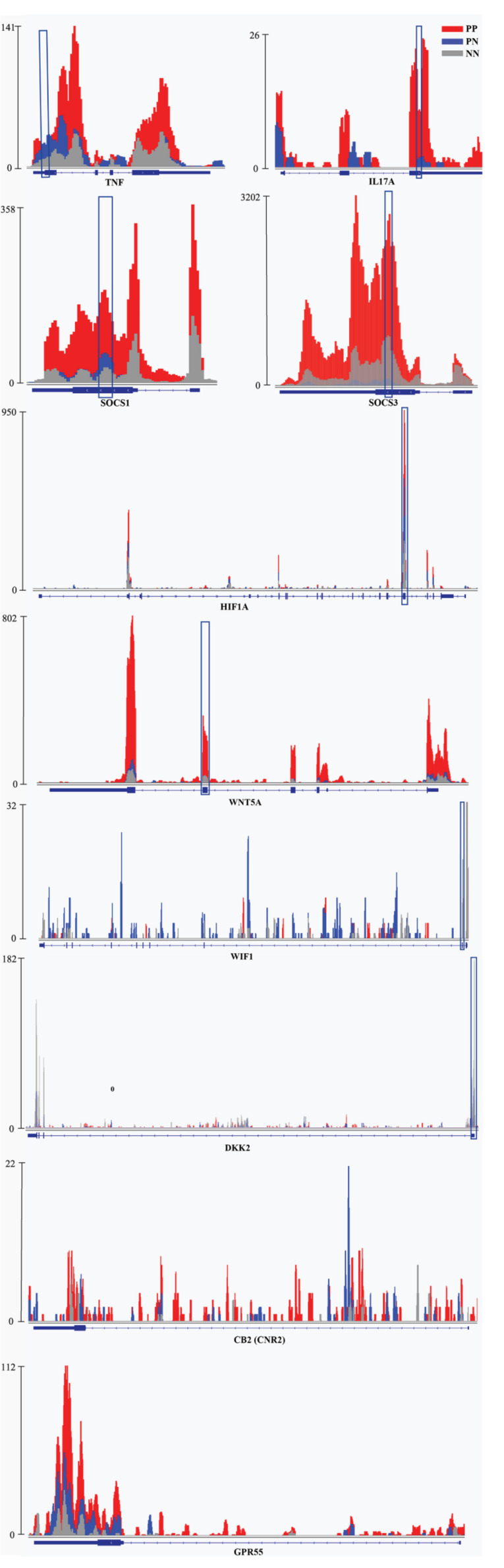
Examples of genes with m^6^A peaks in PP, PN, and NN. Integrative Genomics Viewer (IGV) tracks showed MeRIP-seq reads distribution in TNF, IL-17A, SOCS1/3, HIF1A, WNT5A, WIF1, DKK2, CNR2, and GPR55 mRNA of PP, PN, and NN. Different colors illustrate the accumulation of m^6^A-IP reads. The blue squares indicate the verified sequence fragments.

Kyoto Encyclopedia of Genes and Genomes (KEGG) pathway analysis of DMRs was also conducted for both hypermethylated and hypomethylated genes. This analysis showed that, compared with NN or PN, hypermethylated DMRs in PP were mainly associated with cytokine–cytokine receptor interaction (Figure 2D, Supplementary Figure 5E, and Supplementary Data 4); hypomethylated DMRs in PP were mainly associated with the Wnt signaling pathway (Figure 2E, Supplementary Figure 5F, and Supplementary Data 4). We used quantitative reverse-transcription PCR (RT-qPCR) to validate the important genes WNT5A, DIF1, and DKK2 in Wnt signaling pathway, as well as TNF, IL17A, HIF1A, and SOCS1/3, which are associated with the pathophysiology of psoriasis; all of them showed significant enrichment in immunoprecipitation (IP) pull-down samples (Figure 3 and Supplementary Figure 6). In addition to these shared pathways, DMRs in the PP vs. NN and PP vs. PN comparisons were also associated with some specific pathways. For example, in PP vs. NN, hypermethylated DMRs in PP were specifically associated with olfactory transduction (Figure 2D and Supplementary Data 4), which may explain why psoriasis patients have an abnormal sense of smell (Aydın et al., 2016); meanwhile, hypomethylated DMRs in PP were specifically associated with the MAPK signaling pathway (Figure 2E and Supplementary Data 4), which is involved in almost every aspect of psoriasis vulgaris, including keratinocyte proliferation, differentiation, migration, T-helper-cell differentiation, and angiogenesis (Mavropoulos et al., 2013). In the PP vs. PN comparison, DMRs were specifically associated with the cell cycle and gap junction (Supplementary Figures 5E,F and Supplementary Data 4). Besides, from the comparison with NN, DMRs in PN were mainly associated with vascular smooth muscle contraction and osteoclast differentiation (Supplementary Figures 5G,H and Supplementary Data 4).

### Relationship Between m^6^A Peaks and mRNA Level

**FIGURE 4:**
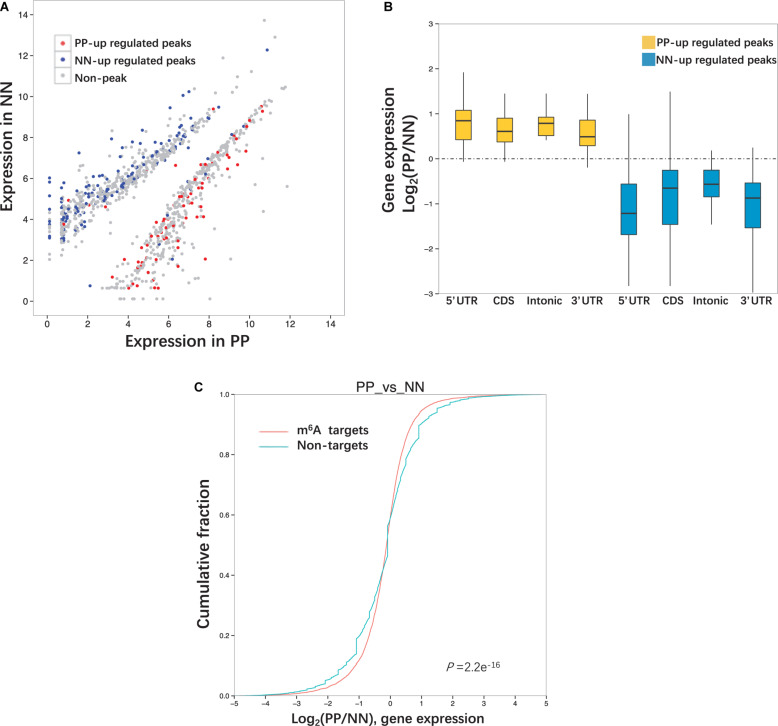
Relationship between m^6^A peaks and mRNA level. **(A)** Differentially expressed genes (transcripts) in PP and NN. Genes contained up-regulated m^6^A peaks are highlighted in red and genes contained down- regulated m^6^A peaks are highlighted in blue. **(B)** The ratio of gene expression levels in PP and NN samples containing up-regulated m^6^A peaks. Genes are divided into four categories (5′UTR, CDS, Intonic, 3′UTR) according to the peak positions. **(C)** Cumulative distribution of mRNA expression changes between PP and NN for m^6^A -modified genes (red) and non-target genes (green). *P*-values are calculated by two-sided Mann–Whitney test.

We next performed MeRIP-Seq and RNA-Seq combined analysis to explore whether the extent of m^6^A methylation was associated with the mRNA level of the differentially expressed genes (DEGs). Specifically, upon comparison with NN, 511 highly expressed DEGs were identified in PP, of which 116 (22.7%) transcripts were modified by m^6^A, 87.9% (102/116) of which were hypermethylated. Meanwhile, 773 transcripts expressed at lower levels were identified in PP, of which 340 (44.0%) were modified by m^6^A, 97.6% (332/340) of which were hypomethylated (Table 2, Figure 4A, and Supplementary Data 5). The comparison of PP vs. PN and PN vs. NN showed a similar pattern to the comparisons of PP vs. NN (Table 2, Supplementary Figures 7A,B, and Supplementary Data 5). Based on these results, we estimated that 19.3–48.4% of the DEGs (transcripts) were chemically modified by m^6^A. In the PP vs. NN and PP vs. PN comparisons, the proportion of transcripts expressed at lower levels that were modified by m^6^A was higher than that of highly expressed transcripts (46.1 vs. 23.7%, *P* < 0.001), suggesting that transcripts expressed at lower levels were more preferentially modified by m^6^A. These results also showed that the upregulation of gene expression was often accompanied by the upregulation of m^6^A methylation (r = 1, *P* < 0.05). Furthermore, we classified these m^6^A-containing DEGs by their peak position and examined how their mRNA expression levels correlated with the locations of m^6^A peaks. Our analysis showed that, irrespective of whether the m^6^A peaks were located at a 5′UTR, CDS, intron, or 3′UTR, their extent of methylation was always positively correlated with the mRNA expression levels (*P* < 0.001) (Figure 4B, Supplementary Figure 8, and Supplementary Data 6).

**TABLE 2:** Relationship between m^6^A peaks and mRNA level.

	PP vs. NN	PP vs. PN	PN vs. NN
Highly expressed DEGs (transcripts)	511	1,330	409
Highly expressed transcripts modified by m^6^A	22.7% (116/511)	24.1% (320/1,330)	23.2% (95/409)
Hypermethylated highly expressed transcripts	87.9% (102/116)	81.9% (262/320)	94.7% (90/95)
Lower expressed DEGs (transcripts)	773	696	498
Lower expressed transcripts modified by m^6^A	44.0% (340/773)	48.4% (337/696)	19.3% (96/498)
Hypomethylated lower expressed transcripts	97.6% (332/340)	94.3% (318/337)	91.7% (88/96)

*DEGs, differentially expressed genes.*

To compare the mRNA expression levels of methylated and unmethylated transcripts, we divided the whole transcriptome genes according to whether they were modified by m^6^A. However, we found that, in the PP vs. NN comparison, among m^6^A methylated genes, more were upregulated than downregulated; among non-m^6^A methylated genes, more were downregulated than upregulated. This trend was consistent with the PP vs. PN and PN vs. NN comparisons (Figure 4C and Supplementary Figure 9).

## Discussion

Extensive m^6^A-related studies have been performed in malignant tumors, nervous system diseases, hematopoietic diseases, metabolic diseases, viral diseases, and injuries, but no reports have been published on m^6^A profiling in patients with psoriasis vulgaris (Brocard et al., 2017; Zhang et al., 2017; Sun et al., 2019). In this study, two sequencing libraries, namely, m^6^A-Seq library (IP) and RNA-Seq library (input), were constructed for high-throughput MeRIP-Seq to examine transcriptome-wide m^6^A methylation patterns in a total of 12 skin samples, four each from PP, PN, and NN biopsies. Our data showed that m^6^A is highly conserved across psoriasis vulgaris and healthy controls. Nevertheless, there are differences among PP, PN, and NN, with ∼5,624 fewer m^6^A peaks identified in PP samples than in PN samples.

m^6^A-modified nucleotides were previously shown to be widely distributed in human tissues including the liver, kidney, brain, lung, and heart (Dominissini et al., 2012; He et al., 2019; Lan et al., 2019; Mathiyalagan et al., 2019). Here, we detected 1.021, 1.254, and 1.176 m^6^A peaks per gene in skin samples of PP, PN, and NN, respectively. These results further affirm that m^6^A is a universal form of RNA modification in human tissues. However, the levels of m^6^A methylation in skin samples of PP, PN, and NN were lower than the previous estimations of approximately ∼1.7 m^6^A residues per gene in a human hepatocellular carcinoma cell line (HepG2) and ∼3 m^6^A residues per average mRNA transcript in mammalian cells (Dominissini et al., 2012). The differences in proportions of m^6^A-modified transcripts may be due to the different tissue types. The pattern of adenosine methylation in mRNAs of PP, PN, and NN is consistent with that reported in mouse brain, in that a few of the m^6^A peaks are clustered together while most of them are single (Meyer et al., 2012). However, the proportions of single peaks of PP, PN, and NN (59.68, 52.95, and 55.25%) are even higher than that of mouse brain (46%) (Meyer et al., 2012). We assumed that this difference might be associated with the different tissue types and gene structure characteristics.

Comparative analysis among groups showed that PP and NN contained the fewest shared m^6^A peaks, while PN and NN carried the most. In addition, DMR analysis revealed that the greatest differences were observed in the PP vs. NN and PP vs. PN comparisons, instead of PN and NN, suggesting that the difference in m^6^A methylation between PP and NN was greater than that between PN and NN. Compared with PN or NN, the number of hypomethylated DMRs in PP was much greater than that of hypermethylated DMRs, suggesting that DMRs in PP were preferentially hypomethylated. These hypermethylated m^6^A peaks in PP were mainly enriched in CDSs and 3′UTRs, which is consistent with previous studies (Meyer et al., 2012). However, the hypomethylated m^6^A peaks in PP were not only enriched in CDSs and 3′UTRs, but also highly enriched in 5′UTRs. Because 5′UTRs play a major role in controlling translation efficiency and shaping the cellular proteome (Hinnebusch et al., 2016), we assume that the hypomethylated DMRs in PP may be involved in protein translation and shaping.

GO analysis revealed that, compared with PN or NN, hypermethylated DMRs in PP were mainly linked to response-associated terms (e.g., response to other organisms, inflammatory response, and response to external/biotic stimulus) and cytokine production and regulation. Because psoriasis is often triggered by internal or external environmental stimuli and persists due to cross-talk between keratinocytes and immunocytes, which mediates the production of cytokines, chemokines, and growth factors (Hugh and Weinberg, 2018), it is suggested that m^6^A methylation may be involved in the important pathogenic processes of psoriasis, including triggering the disease and maintaining inflammation. Distinct from hypermethylated DMRs in PP, the hypomethylated DMRs in PP were mainly associated with development-related terms, suggesting that hypomethylated DMRs in PP may participate in development-related processes. In addition, compared with NN, highly methylated DMRs in PP were specifically enriched in the G-protein-coupled receptor signaling pathway. Studies have reported that cannabinoid type 2 receptor (CB2) and G-protein-coupled receptor 55 (GPR55), as G-protein-coupled receptors, were both increased in psoriasis vulgaris and could attenuate oxidative stress and act in an anti-inflammatory process (Ambrożewicz et al., 2018; Figure 3). Another study reported that cannabinoid receptors control the proliferation/differentiation and immune activity of keratinocytes (Tóth et al., 2019). While both CB2 and GPR55 were modified by m^6^A, there was no significant difference among PP, PN and NN in our study (Figure 3). Meanwhile, hypomethylated DMRs in PP were particularly associated with the polyol metabolic process, which is one of the major biochemical pathways involved in the development of diabetic macroangiopathy and peripheral neuropathy (Katakami, 2018). It has been shown that there is a significant correlation between psoriasis/diabetes and the polyol metabolic process (Mamizadeh et al., 2019), so we consider that these hypomethylated DMRs in PP may provide a clue to explain why psoriasis is related to diabetes.

Interestingly, KEGG pathway analysis revealed that, compared with NN, highly methylated DMRs in PP were mainly associated with olfactory receptor genes. Ahn et al. (2016) reported that modules linked to psoriasis were mainly associated with olfactory signaling, as assessed by RNA-Seq. Olfactory receptors are known to be expressed not only in nasal tissue but also in skin tissue and specifically in keratinocytes, dendritic cells, and melanocytes (Ahn et al., 2016). In addition, Aydın et al. (2016) found that the olfactory function of patients with psoriasis was significantly worse than that of healthy controls (*P* < 0.001). These results suggest that m^6^A methylation may be involved in the mechanism of anosmia in psoriasis vulgaris. In addition, our studies showed that hypomethylated transcripts in PP were mainly associated with the Wnt signaling pathway. The Wnt gene family is known to encode a set of highly conserved secreted signaling proteins that participate in and control cell differentiation, cell proliferation, and immune-mediated inflammatory cascade in psoriasis vulgaris (Gudjonsson et al., 2010; Dou et al., 2017; Wang et al., 2017a). This suggests that m^6^A methylation may control the critical pathogenic processes in psoriasis vulgaris, including cell differentiation, cell proliferation, and immune-mediated inflammation, by modifying expression of the Wnt gene family (Irrera et al., 2020). Besides, we found that IL-17A and TNF-α, two of the key genes of the TNF-α/IL-23/Th17 axis in psoriasis, are upregulated > 20-fold and > 3.5-fold in m^6^A methylation levels in PP vs. NN, respectively (Figure 3). This axis is widely regarded as the core process of the pathogenesis of psoriasis vulgaris, which could induce the proliferation of keratinocytes and form the inflammatory plaque of psoriasis vulgaris (Rendon and Schäkel, 2019). The rapid and efficient therapeutic response of various monoclonal antibodies against TNF-α or IL-17A strongly supports the TNF-α/IL-23/Th17 axis as being a key factor for the expansion of inflammation and the aggravation of skin lesions in psoriasis vulgaris (Rendon and Schäkel, 2019). Therefore, these findings support the notion that m^6^A may be a critical denominator that controls keratinocyte proliferation, cell differentiation, and inflammation in psoriasis vulgaris.

RNA-Seq data were used for the combined analysis of m^6^A peaks and mRNA level. Upon comparison with PN or NN, about 20% of the highly expressed genes in PP (PP-high) were chemically modified by m^6^A, while about 40% of the genes expressed at lower levels in PP (PP-low) were chemically modified by m^6^A, which was close to the findings in a previous report (over one-third) on the human brain (Dominissini et al., 2012). The estimated difference between PP-high and PP-low suggested that genes expressed at lower levels were preferentially modified by m^6^A. We extended this analysis to the whole transcriptome. However, among genes expressed at a low level in PP, the proportion of m^6^A targets was smaller than that of non-m^6^A targets; meanwhile, among highly expressed genes in PP, the proportion of m^6^A targets was greater than that of non-m^6^A targets. The reason for the results being opposite between the two analyses may be the different data ranges. The former analysis only analyzed DEGs, while the latter analysis analyzed the whole transcriptome. In previous studies on *A. thaliana*, most transcripts with a low expression level were more likely modified by m^6^A in both leaf and flower chloroplasts (Wan et al., 2015; Wang et al., 2017c). However, the root amyloplast presented the methylation feature that the moderately expressed transcripts were more likely to be methylated, and those expressed at the two extremes were less methylated by m^6^A (Wan et al., 2015; Wang et al., 2017c). These results indicated that the relationship between the level of mRNA and whether there is m^6^A modification differs among different species, organs and tissues. Furthermore, we analyzed the relationship between m^6^A level and mRNA level, and found that the upregulation of gene expression was often accompanied by the upregulation of m^6^A methylation regardless of the peak position, suggesting a possible positive relationship between the extent of m^6^A methylation and the mRNA levels. Actually, many researches have revealed that m^6^A level was positively or negatively correlated with mRNA level (Zhao et al., 2017). Mostly, m^6^A methylation was shown to be negatively correlated with mRNA level when m^6^A methylation was linked to accelerate degradation of target transcripts (Liu et al., 2014). For example, silencing of m^6^A writers (METTL3, METTL14, or WTAP) in mammalian cells has been shown to lead to increases in the abundance of their respective target transcripts (Liu et al., 2014). Overexpression of m^6^A reader (YTHDF2) in human embryonic kidney 293T cells has been shown to lead to decreases in the abundance of the target transcripts (Wu et al., 2020). Besides, m^6^A methylation was shown to be positively correlated with mRNA level when m^6^A methylation was linked to enhance mRNA expression (Zhao et al., 2017). For example, METTL3-mediated m^6^A methylation enhanced ZMYM1 mRNA expression in gastric cancer (Yue et al., 2019). The role of m^6^A methylation in transcriptional regulation needs to be elucidated by further studies in the future.

## Data Availability Statement

The datasets generated for this study can be found in the online repositories. The names of the repository/repositories and accession number(s) can be found below: https://www.ncbi.nlm.nih.gov/geo/query/acc.cgi?acc=GSE155702.

## Ethics Statement

The studies involving human participants were reviewed and approved by the Ethics Committee of Peking Union Medical College Hospital. The patients/participants provided their written informed consent to participate in this study.

## Author Contributions

Y-NW conceived the project, performed the experiments, performed data analyses, and wrote the manuscript. H-ZJ reviewed the manuscript and made substantial contributions to the drafting process. All authors agreed to be accountable for the content of the work.

## Conflict of Interest

The authors declare that the research was conducted in the absence of any commercial or financial relationships that could be construed as a potential conflict of interest.

## Footnotes

1 www.geneontology.org

2 www.genome.jp/kegg

## References

[B1] Ahn R. Gupta R. Lai K. Chopra N. Arron S. T. Liao W. (2016). Network analysis of psoriasis reveals biological pathways and roles for coding and long non-coding RNAs. *BMC Genomics* 17:841. 10.1186/s12864-016-3188-y 27793094 PMC5084355

[B2] Ambrożewicz E. Wójcik P. Wroński A. Łuczaj W. Jastrząb A. Žarković N. (2018). Pathophysiological Alterations of Redox Signaling and Endocannabinoid System in Granulocytes and Plasma of Psoriatic Patients. *Cells* 7:159. 10.3390/cells7100159 30301214 PMC6210326

[B3] Aydın E. Tekeli H. Karabacak E. Altunay ÝK. Aydın Ç Çerman A. A. (2016). Olfactory functions in patients with psoriasis vulgaris: correlations with the severity of the disease. *Arch. Dermatol. Res.* 308 409–414. 10.1007/s00403-016-1662-7 27299882

[B4] Bi Z. Liu Y. Zhao Y. Yao Y. Wu R. Liu Q. (2019). A dynamic reversible RNA N(6) -methyladenosine modification: current status and perspectives. *J. Cell Physiol.* 234 7948–7956. 10.1002/jcp.28014 30644095

[B5] Boehncke W. H. Schon M. P. (2015). Psoriasis. *Lancet* 386 983–994. 10.1016/s0140-6736(14)61909-7 26025581

[B6] Bovenschen H. J. van de Kerkhof P. C. van Erp P. E. Woestenenk R. Joosten I. Koenen H. J. (2011). Foxp3+ regulatory T cells of psoriasis patients easily differentiate into IL-17A-producing cells and are found in lesional skin. *J. Invest. Dermatol.* 131 1853–1860. 10.1038/jid.2011.139 21654831

[B7] Brocard M. Ruggieri A. Locker N. (2017). m6A RNA methylation, a new hallmark in virus-host interactions. *J. Gen. Virol.* 98 2207–2214. 10.1099/jgv.0.000910 28869001

[B8] Church C. Moir L. McMurray F. Girard C. Banks G. T. Teboul L. (2010). Overexpression of Fto leads to increased food intake and results in obesity. *Nat. Genet.* 42 1086–1092. 10.1038/ng.713 21076408 PMC3018646

[B9] Dominissini D. Moshitch-Moshkovitz S. Salmon-Divon M. Amariglio N. Rechavi G. (2013). Transcriptome-wide mapping of N(6)-methyladenosine by m(6)A-seq based on immunocapturing and massively parallel sequencing. *Nat. Protoc.* 8 176–189. 10.1038/nprot.2012.148 23288318

[B10] Dominissini D. Moshitch-Moshkovitz S. Schwartz S. Salmon-Divon M. Ungar L. Osenberg S. (2012). Topology of the human and mouse m6A RNA methylomes revealed by m6A-seq. *Nature* 485 201–206. 10.1038/nature11112 22575960

[B11] Dou J. Zhang L. Xie X. Ye L. Yang C. Wen L. (2017). Integrative analyses reveal biological pathways and key genes in psoriasis. *Br. J. Dermatol.* 177 1349–1357. 10.1111/bjd.15682 28542811

[B12] Engel M. Chen A. (2018). The emerging role of mRNA methylation in normal and pathological behavior. *Genes Brain Behav.* 17:e12428. 10.1111/gbb.12428 29027751

[B13] Frye M. Harada B. T. Behm M. He C. (2018). RNA modifications modulate gene expression during development. *Science* 361 1346–1349. 10.1126/science.aau1646 30262497 PMC6436390

[B14] Fu Y. Dominissini D. Rechavi G. He C. (2014). Gene expression regulation mediated through reversible m(6)A RNA methylation. *Nat. Rev. Genet.* 15 293–306. 10.1038/nrg3724 24662220

[B15] Greb J. E. Goldminz A. M. Elder J. T. Lebwohl M. G. Gladman D. D. Wu J. J. (2016). Psoriasis. *Nat. Rev. Dis. Prim.* 2:16082. 10.1038/nrdp.2016.82 27883001

[B16] Gudjonsson J. E. Johnston A. Stoll S. W. Riblett M. B. Xing X. Kochkodan J. J. (2010). Evidence for altered Wnt signaling in psoriatic skin. *J. Invest. Dermatol.* 130 1849–1859. 10.1038/jid.2010.67 20376066 PMC2886156

[B17] Hammitzsch A. Tallant C. Fedorov O. O’Mahony A. Brennan P. E. Hay D. A. (2015). CBP30, a selective CBP/p300 bromodomain inhibitor, suppresses human Th17 responses. *Proc. Natl. Acad. Sci. U.S.A.* 112 10768–10773. 10.1073/pnas.1501956112 26261308 PMC4553799

[B18] He C. (2010). Grand challenge commentary: RNA epigenetics? *Nat. Chem. Biol.* 6 863–865. 10.1038/nchembio.482 21079590

[B19] He Y. Xing J. Wang S. Xin S. Han Y. Zhang J. (2019). Increased m6A methylation level is associated with the progression of human abdominal aortic aneurysm. *Ann. Transl. Med.* 7:797. 10.21037/atm.2019.12.65 32042813 PMC6989874

[B20] Heinz S. Benner C. Spann N. Bertolino E. Lin Y. C. Laslo P. (2010). Simple combinations of lineage-determining transcription factors prime cis-regulatory elements required for macrophage and B cell identities. *Mol. Cell* 38 576–589. 10.1016/j.molcel.2010.05.004 20513432 PMC2898526

[B21] Hinnebusch A. G. Ivanov I. P. Sonenberg N. (2016). Translational control by 5’-untranslated regions of eukaryotic mRNAs. *Science* 352 1413–1416. 10.1126/science.aad9868 27313038 PMC7422601

[B22] Hu Y. Zhu Y. Lian N. Chen M. Bartke A. Yuan R. (2019). Metabolic syndrome and skin diseases. *Front. Endocrinol.* 10:788. 10.3389/fendo.2019.00788 31824416 PMC6880611

[B23] Hugh J. M. Weinberg J. M. (2018). Update on the pathophysiology of psoriasis. *Cutis* 102 6–12. 30566550

[B24] Irrera N. Bitto A. Vaccaro M. Mannino F. Squadrito V. Pallio G. (2020). PDRN, a Bioactive natural compound, ameliorates imiquimod-induced psoriasis through NF-κB Pathway Inhibition and Wnt/β-catenin signaling modulation. *Int. J. Mol. Sci.* 21:1215. 10.3390/ijms21041215 32059361 PMC7072802

[B25] Katakami N. (2018). Mechanism of development of atherosclerosis and cardiovascular disease in diabetes Mellitus. *J. Atheroscler. Thromb.* 25 27–39. 10.5551/jat.RV17014 28966336 PMC5770221

[B26] Kim D. Langmead B. Salzberg S. L. (2015). HISAT: a fast spliced aligner with low memory requirements. *Nat. Methods* 12 357–360. 10.1038/nmeth.3317 25751142 PMC4655817

[B27] Lan Q. Liu P. Y. Haase J. Bell J. L. Hüttelmaier S. Liu T. (2019). The critical role of RNA m(6)A methylation in cancer. *Cancer Res.* 79 1285–1292. 10.1158/0008-5472.Can-18-2965 30894375

[B28] Liu J. Yue Y. Han D. Wang X. Fu Y. Zhang L. (2014). A METTL3-METTL14 complex mediates mammalian nuclear RNA N6-adenosine methylation. *Nat. Chem. Biol.* 10 93–95. 10.1038/nchembio.1432 24316715 PMC3911877

[B29] Mamizadeh M. Tardeh Z. Azami M. (2019). The association between psoriasis and diabetes mellitus: a systematic review and meta-analysis. *Diabetes Metab. Syndr.* 13 1405–1412. 10.1016/j.dsx.2019.01.009 31336500

[B30] Martin M. (2011). Cutadapt removes adapter sequences from high-throughput sequencing reads. *EMBnet J* 17 10–12.

[B31] Mathiyalagan P. Adamiak M. Mayourian J. Sassi Y. Liang Y. Agarwal N. (2019). FTO-Dependent N(6)-methyladenosine regulates cardiac function during remodeling and repair. *Circulation* 139 518–532. 10.1161/circulationaha.118.033794 29997116 PMC6400591

[B32] Mavropoulos A. Rigopoulou E. I. Liaskos C. Bogdanos D. P. Sakkas L. I. (2013). The role of p38 MAPK in the aetiopathogenesis of psoriasis and psoriatic arthritis. *Clin. Dev. Immunol.* 2013:569751. 10.1155/2013/569751 24151518 PMC3787653

[B33] Meyer K. D. Saletore Y. Zumbo P. Elemento O. Mason C. E. Jaffrey S. R. (2012). Comprehensive analysis of mRNA methylation reveals enrichment in 3’ UTRs and near stop codons. *Cell* 149 1635–1646. 10.1016/j.cell.2012.05.003 22608085 PMC3383396

[B34] Pan Y. Ma P. Liu Y. Li W. Shu Y. (2018). Multiple functions of m(6)A RNA methylation in cancer. *J. Hematol. Oncol.* 11:48. 10.1186/s13045-018-0590-8 29587823 PMC5870302

[B35] Pasquali L. Srivastava A. Meisgen F. Das Mahapatra K. Xia P. Xu Landén N. (2019). The keratinocyte transcriptome in psoriasis: pathways related to immune responses. cell cycle and keratinization. *Acta Derm Venereol.* 99 196–205. 10.2340/00015555-3066 30320872

[B36] Pollock R. A. Abji F. Gladman D. D. (2017). Epigenetics of psoriatic disease: a systematic review and critical appraisal. *J. Autoimmun.* 78 29–38. 10.1016/j.jaut.2016.12.002 27965059

[B37] Rendon A. Schäkel K. (2019). Psoriasis pathogenesis and treatment. *Int. J. Mol. Sci.* 20:1475. 10.3390/ijms20061475 30909615 PMC6471628

[B38] Shen L. Shao N. Y. Liu X. Maze I. Feng J. Nestler E. J. (2013). diffReps: detecting differential chromatin modification sites from ChIP-seq data with biological replicates. *PLoS One* 8:e65598. 10.1371/journal.pone.0065598 23762400 PMC3677880

[B39] Sun T. Wu R. Ming L. (2019). The role of m6A RNA methylation in cancer. *Biomed. Pharmacother.* 112:108613. 10.1016/j.biopha.2019.108613 30784918

[B40] Thorvaldsdóttir H. Robinson J. T. Mesirov J. P. (2013). Integrative Genomics Viewer (IGV): high-performance genomics data visualization and exploration. *Brief Bioinform.* 14 178–192. 10.1093/bib/bbs017 22517427 PMC3603213

[B41] Tóth K. F. Ádám D. Bíró T. Oláh A. (2019). Cannabinoid signaling in the skin: therapeutic potential of the “C(ut)annabinoid” System. *Molecules* 24:918. 10.3390/molecules24050918 30845666 PMC6429381

[B42] Trapnell C. Roberts A. Goff L. Pertea G. Kim D. Kelley D. R. (2012). Differential gene and transcript expression analysis of RNA-seq experiments with TopHat and Cufflinks. *Nat. Protoc.* 7 562–578. 10.1038/nprot.2012.016 22383036 PMC3334321

[B43] Wan Y. Tang K. Zhang D. Xie S. Zhu X. Wang Z. (2015). Transcriptome-wide high-throughput deep m(6)A-seq reveals unique differential m(6)A methylation patterns between three organs in Arabidopsis thaliana. *Genome Biol.* 16:272. 10.1186/s13059-015-0839-2 26667818 PMC4714525

[B44] Wang W. Yu X. Wu C. Jin H. (2017a). IL-36γ inhibits differentiation and induces inflammation of keratinocyte via Wnt signaling pathway in psoriasis. *Int. J. Med. Sci.* 14 1002–1007. 10.7150/ijms.20809 28924372 PMC5599924

[B45] Wang X. Huang J. Zou T. Yin P. (2017b). Human m(6)A writers: two subunits, 2 roles. *RNA Biol.* 14 300–304. 10.1080/15476286.2017.1282025 28121234 PMC5367249

[B46] Wang Y. N. Yu C. Y. Jin H. Z. (2020). RNA N(6)-Methyladenosine Modifications and the Immune Response. *J. Immunol. Res.* 2020:6327614. 10.1155/2020/6327614 32411802 PMC7204177

[B47] Wang Z. Tang K. Zhang D. Wan Y. Wen Y. Lu Q. (2017c). High-throughput m6A-seq reveals RNA m6A methylation patterns in the chloroplast and mitochondria transcriptomes of Arabidopsis thaliana. *PLoS One* 12:e0185612. 10.1371/journal.pone.0185612 29131848 PMC5683568

[B48] World Health Organization. (2014). *Resolution WHA67.9 Psoriasis Sixty-Seventh World Health Assembly, Resolutions and Decisions.* Geneva: WHO.

[B49] Wu C. Chen W. He J. Jin S. Liu Y. Yi Y. (2020). Interplay of m(6)A and H3K27 trimethylation restrains inflammation during bacterial infection. *Sci. Adv.* 6:eaba0647. 10.1126/sciadv.aba0647 32875102 PMC7438091

[B50] Yue B. Song C. Yang L. Cui R. Cheng X. Zhang Z. (2019). METTL3-mediated N6-methyladenosine modification is critical for epithelial-mesenchymal transition and metastasis of gastric cancer. *Mol. Cancer* 18:142. 10.1186/s12943-019-1065-4 PMC6790244 31607270

[B51] Zhang C. Chen Y. Sun B. Wang L. Yang Y. Ma D. (2017). m(6)A modulates haematopoietic stem and progenitor cell specification. *Nature* 549 273–276. 10.1038/nature23883 28869969

[B52] Zhang Y. Liu T. Meyer C. A. Eeckhoute J. Johnson D. S. Bernstein B. E. (2008). Model-based analysis of ChIP-Seq (MACS). *Genome Biol.* 9:R137. 10.1186/gb-2008-9-9-r137 18798982 PMC2592715

[B53] Zhao B. S. Roundtree I. A. He C. (2017). Post-transcriptional gene regulation by mRNA modifications. *Nat. Rev. Mol. Cell Biol.* 18 31–42. 10.1038/nrm.2016.132 27808276 PMC5167638

