# Supplementary material for: Transcriptome-Wide m6A Methylation in Skin Lesions From Patients With Psoriasis Vulgaris
Source: Front Cell Dev Biol. 2020 Nov 5;8:591629. doi: 10.3389/fcell.2020.591629 (PMC7674922; doi:10.3389/fcell.2020.591629)
Supplement: Supplementary file 1 [file Data_Sheet_1.docx]

Supplementary Material

# Supplementary Figures and Tables

# Supplementary Table 1 Clinical characteristics of four patients with psoriasis vulgaris

| **Patient No.** | **Gender/age(years)** | **Disease course (years)** | **PASI score** | **Family history** | **Original site** |
| --- | --- | --- | --- | --- | --- |
| 1 | M/21 | 1 | 10.8 | - | Upper extremity |
| 2 | F/17 | 1 | 16.7 | - | Back |
| 3 | F/14 | 3 | 10.8 | - | Lower extremity |
| 4 | M/32 | 8 | 15.6 | - | Back |

PASI: Psoriasis Area and Severity Index

**Supplementary Table 2 Primers used for quantitative real-time PCR**

| **Gene** | **Forward and reverse primer** |
| --- | --- |
| WNT5A | F:5’ CGTGGGTGGATTAATTTGGA 3’ |
|  | R: 5’ TGTTGATTGACTGCGCTTCT 3’ |
| TNF | F:5’ GGACACCATGAGCACTGAAA 3’ |
|  | R: 5’ AAGAGGCTGAGGAACAAGCA 3’ |
| WIF1 | F:5’ ATCCTCCTGTGCCTGCTG 3’ |
|  | R: 5’ ACTCTTGCCTGGTGAGCATC 3’ |
| DKK2 | F:5’ GAGCACTGTGTCCTGCAAAG 3’ |
|  | R: 5’ GCATCAGCTCCTTCTCCTTC 3’ |
| HIF1A | F:5’ CACTACCACTGCCACCACTG 3’ |
|  | R: 5’ TGGGTAGGAGATGGAGATGC 3’ |
| IL17A | F:5’ TCCGGCTGGAGAAGATACTG 3’ |
|  | R: 5’ CCAGAGCTCTTAGGCCACAT 3’ |
| SOCS1 | F:5’ GGATGGTAGCACACAACCAG 3’ |
|  | R: 5’ GAGGAGGAGGAAGAGGAGGA 3’ |
| SOCS3 | F:5’ CTTCGACTGCGTGCTCAAG 3’ |
|  | R: 5’ CGGAGGAGGGTTCAGTAGGT 3’ |

**Supplementary Table 3 Summary of sequence data and read alignment statistics**

|  | **Sample** | **Raw reads** | **Clean reads^†^** | **Reads uniquely mapped to genome** | **Total reads uniquely mapped (%)** |
| --- | --- | --- | --- | --- | --- |
| m^6^A-IP^‡^ | PP | 68,678,097 | 68,456,993 | 57,334,158 | 83.11% |
| (m^6^A-Seq) | PN | 62,087,334 | 61,881,403 | 45,130,912 | 72.65% |
|  | NN | 62,121,508 | 61,930,833 | 49,749,336 | 80.04% |
| m^6^A-Input | PP | 36,624,408 | 36,591,148 | 33,190,954 | 77.80% |
| (m^6^A-Seq) | PN | 44,576,854 | 43,163,746 | 31,169,867 | 90.78% |
|  | NN | 38,514,904 | 38,492,302 | 35,335,938 | 91.85% |

Note: **^†^**Clean reads indicate reads after filtering adaptors and low-quality reads, and random sampling; ^‡^IP, immunoprecipitation.

**Supplementary Table 4 m^6^A peak density of transcripts with FPKM>2**

| **Group** | **Peaks** | **Genes (FPKM>2)** | **Total length** | **Average peak density (m^6^A sites/gene)** | **Average peak density**  **(m^6^A sites/1k nt)** |
| --- | --- | --- | --- | --- | --- |
| PP | 16,868 | 16,520 | 27,295,965 | 1.021065375 | 0.6179668 |
| PN | 22,144 | 17,665 | 29,357,796 | 1.253552222 | 0.75428006 |
| NN | 20,408 | 17,358 | 29,073,882 | 1.175711487 | 0.70193585 |

Transcripts are filtered by FPKM value (>2, calculated by Cufflinks6). The total lengths of expressed genes were calculated corresponding to the same criteria for PP, PN, and NN.

**Supplementary Table 5 Number of m^6^A peaks and peak-containing genes among PP, PN, and NN samples**

| **m^6^A peak** | **Hypermethylated**  **in skin lesions(m^6^A peak/gene)** | **Hypomethylated**  **in skin lesions(m^6^A peak/gene)** |
| --- | --- | --- |
| PP vs NN | 1470/1127 | 1719/1113 |
| PP vs PN | 2024/1362 | 2316/1568 |
| PN vs NN | 914/691 | 755/537 |

## Supplementary Figures

**Supplementary Figure 1. RPM of each sample from PP, PN and NN(n = 4).** RPM, reads of peak-containing per million mapped reads; PP, patients with psoriasis vulgaris; PN, uninvolved psoriatic skin; NN, healthy controls.

**Supplementary Figure 2.** Comparison of the number of m6A peaks identified in (A) PP and NN and (B) PN and NN.

**Supplementary Figure 3.** The top five motifs enriched across m^6^A peaks identified from (A) PN and (B) NN.

**Supplementary Figure 4.** The distribution of m^6^A peaks from (A) PN and (B) NN along a metagene.

**Supplementary Figure 5.** The top 10 Gene Ontology terms significantly enriched for the hypermethylated genes in (A) PP vs. PN and (C) PN vs. NN. The top 10 Gene Ontology terms significantly enriched for the hypomethylated genes in (B) PP vs. PN and (D) PN vs. NN. The top 10 significantly enriched pathways for the hypermethylated genes in (E) PP vs. PN and (G) PN vs. NN. The top 10 significantly enriched pathways for the hypomethylated genes in (F) PP vs. PN and (H) PN vs. NN.

**Supplementary Figure 6.** Real-time RT-qPCR validation for eight m6A-containing transcripts (P < 0.05). Eight transcripts enriched in m6A IP were tested. RNA that passed through a beads-only column, which should not bind to any RNA, was treated as the input control for the IP step. Ct values from qPCR on the flow-through from the m6A IP and the m6A IP were expressed as the percent of the input. Two-tailed paired Student’s t test was used.

**Supplementary Figure 7.** Differentially expressed genes (transcripts) in (A) PP and PN and (B) PN and NN. Genes containing upregulated m^6^A peaks are highlighted in red and genes contained downregulated m^6^A peaks are highlighted in blue.

**Supplementary Figure 8.** The proportion of gene expression levels in (A) PP and PN and (B) PN and NN samples containing upregulated m^6^A peaks. Genes are divided into four categories (5′UTR, CDS, intron, 3′UTR) according to the peak positions.

**Supplementary Figure 9.** Cumulative distribution of mRNA expression changes between (A) PP and PN and (B) PN and NN for m^6^A-modified genes (red) and non-target genes (green). P- values were calculated by two-sided Mann–Whitney test.
